# Mobile mechanical signal generator for macrophage polarization

**DOI:** 10.1002/EXP.20220147

**Published:** 2023-04-03

**Authors:** Jiamiao Jiang, Fei Wang, Weichang Huang, Jia Sun, Yicheng Ye, Juanfeng Ou, Meihuan Liu, Junbin Gao, Shuanghu Wang, Dongmei Fu, Bin Chen, Lu Liu, Fei Peng, Yingfeng Tu

**Affiliations:** ^1^ NMPA Key Laboratory for Research and Evaluation of Drug Metabolism & Guangdong Provincial Key Laboratory of New Drug Screening, School of Pharmaceutical Sciences Southern Medical University Guangzhou China; ^2^ Department of Critical Care Medicine, Dongguan Institute of Respiratory and Critical Care Medicine Affiliated Dongguan Hospital Southern Medical University Dongguan China; ^3^ The Laboratory of Clinical Pharmacy The Sixth Affiliated Hospital of Wenzhou Medical University, The People's Hospital of Lishui Lishui China; ^4^ School of Materials Science and Engineering Sun Yat‐Sen University Guangzhou China

**Keywords:** low Reynolds number, macrophage polarization, magnetic self‐assembly, mechanical stimulation, micro/nanorobots

## Abstract

The importance of mechanical signals in regulating the fate of macrophages is gaining increased attention recently. However, the recently used mechanical signals normally rely on the physical characteristics of matrix with non‐specificity and instability or mechanical loading devices with uncontrollability and complexity. Herein, we demonstrate the successful fabrication of self‐assembled microrobots (SMRs) based on magnetic nanoparticles as local mechanical signal generators for precise macrophage polarization. Under a rotating magnetic field (RMF), the propulsion of SMRs occurs due to the elastic deformation via magnetic force and hydrodynamics. SMRs perform wireless navigation toward the targeted macrophage in a controllable manner and subsequently rotate around the cell for mechanical signal generation. Macrophages are eventually polarized from M0 to anti‐inflammatory related M2 phenotypes by blocking the Piezo1‐activating protein‐1 (AP‐1）‐CCL2 signaling pathway. The as‐developed microrobot system provides a new platform of mechanical signal loading for macrophage polarization, which holds great potential for precise regulation of cell fate.‐

## Graphical abstract

This work describes a novel approach for macrophage polarization utilizing microrobots assembled by magnetic nanoparticles. It not only probed their system for macrophage polarization but also provided an excellent theoretical and experimental demonstration of the magneto‐driven principles of their chain microrobots.

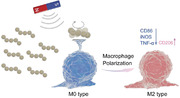

## 1 INTRODUCTION

Macrophages represent a kind of innate immune cell collection that exists widely in tissues and play an important role in fighting infection and maintaining homeostasis.^[^ ^1^ ^]^ As cells with the hallmarks of high heterogeneity and plasticity, macrophages can be polarized with different physiological functions in response to various microenvironmental stimulations.^[^ ^2^, ^3^ ^]^ There are mainly two classic phenotypes of macrophages including classically activated M1 (proinflammatory and antitumor) and alternatively activated M2 (anti‐inflammatory and immunosuppression) phenotypes.^[^ ^4^ ^]^ Normally, the polarization balance of macrophages governs the fate of inflammatory diseases, including cardiovascular disease, cancer, atherosclerosis, wound healing, and others.^[^ ^1^, ^5^ ^]^ With the in‐depth study of the interaction between microenvironment and cells, the importance of mechanical signals acting on macrophages to the tissue homeostasis has been continuously recognized.^[^ ^6^ ^]^ The use of mechanical signals to regulate macrophage polarization for the treatment of inflammation‐related diseases has attracted widespread research interest. By adjusting the physical characteristics of matrix including substrate stiffness,^[^ ^7^, ^8^ ^]^ topography,^[^ ^9^, ^10^ ^]^ and porosity, or using matrix‐provided indirect mechanical force stimulation or mechanical loading devices,^[^ ^11^, ^12^, ^13^, ^14^, ^15^, ^16^, ^17^ ^]^ bulky macrophage polarization can be regulated accordingly. However, these matrices may induce foreign body reaction and the function of the recruited cells such as neutrophils and dendritic cells may change as well due to the lack of selectivity.^[^ ^18^ ^]^ Meanwhile, the uncontrollable effect of degradation‐induced physical property changes of matrix on macrophage polarization cannot be ignored.^[^ ^19^, ^20^ ^]^ For mechanical loading device, it normally has deficiencies of complicated apparatus, cumbersome operation, unequal force and inability to single cell stimulation. Therefore, the design and construction of simple, effective, and stable microenvironment mechanical signal generator are urgently needed.

Micro/nanorobot (MNR) is an active colloid that can convert various energy sources (chemical fuel, magnetic field, light, electric field, and ultrasound) into mechanical motion.^[^ ^21^ ^]^ Due to their autonomous movement and non‐invasiveness, the kinetic energy generated by MNRs results in revolutionary innovations for biomedical applications,^[^ ^22^, ^23^ ^]^ such as cargo delivery,^[^ ^21^ ^]^ cell manipulation,^[^ ^24^, ^25^, ^26^ ^]^ biosensing,^[^ ^27^ ^]^ and precision microsurgery.^[^ ^28^ ^]^ As most widely studied MNRs, magnetically driven microrobots (MMRs), capable of obviating chemical fuel requirements, exhibit high biocompatibility and strong motion controllability, which can perform complex operations and on‐demand tasks in biological fluids.^[^ ^29^, ^30^, ^31^ ^]^ Moreover, the utilization of external magnetic field endows MMRs with a stable and controllable source of mechanical kinetic energy, which is expected to become an effective and stable mechanical signal generator for the regulation of macrophage polarization.

The propulsion of MMRs typically relies on their own chiral structures or nearby surfaces (for hydrodynamic symmetry breaking, which is called “surface walker”).^[^ ^32^ ^]^ However, their applicability is limited due to the complex fabrication and relatively large size of chiral MMRs, and the uncontrollable locomotion behavior of “surface walker” under curved surfaces.^[^ ^31^, ^33^, ^34^ ^]^ Flexible microswimmers based on the self‐assembly of magnetic nanoparticles are possible to generate propulsion by force deformation into a chiral structure under a rotating magnetic field (RMF),^[^ ^35^ ^]^ which are independent with the solid surface and exhibit a controlled motion in viscous fluid. These non‐invasive and easily replicated MMRs, with a chain‐like structure similar to a magnetic paddle, seem like a promising platform for microenvironment mechanical signal loading. Although the self‐assembly chain of magnetic particles in a magnetic field has been well described previously,^[^ ^36^, ^37^ ^]^ and gradient magnetic field driven / nearby surface based chain‐like microrobots have also been reported.^[^ ^34^, ^38^ ^]^ However, the chiral deformation based working principle and motion law of chain MMRs in RMF are not thoroughly understood yet, and its biomedical applications also need to be further expanded.

**FIGURE 1:**
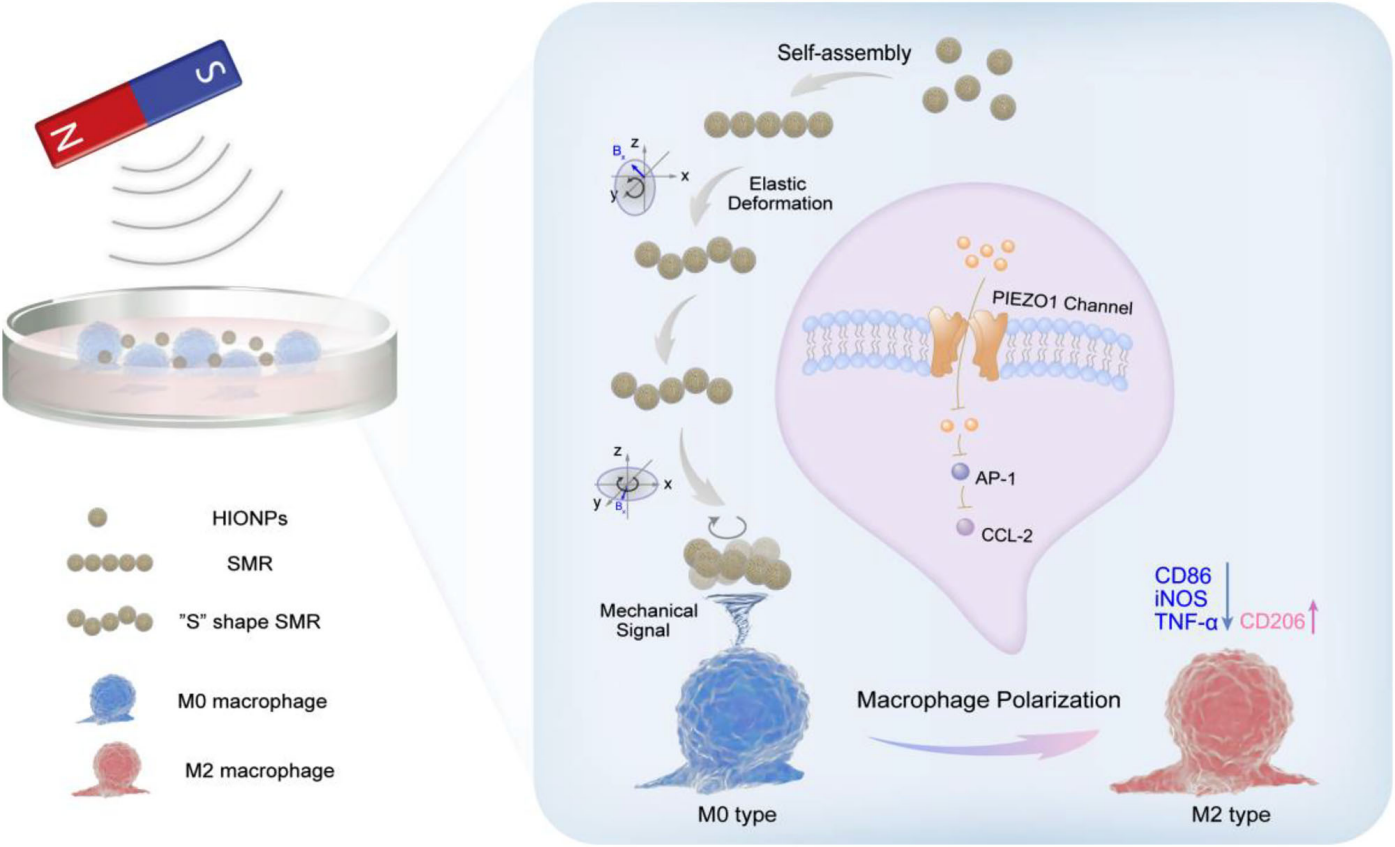
Schematic diagram of SMR used as a novel mechanical signal generator for macrophage polarization.

Herein, flexible chain‐like micro‐robots assembled by hollow iron oxide nanoparticles (HIONPs) were developed as a local mechanical signal generator for macrophage polarization (Figure 1). Mathematical modeling results showed that due to the applied magnetic forces and hydrodynamics, the motion of self‐assembled micro‐robots (SMRs) was generated by elastic deformation induced chiral structure in *x*–*z* rotational plane. Under magnetic field, SMRs exhibited frequency‐related motion behavior including synchronous and asynchronous rotating regions. The deformation period in the low‐frequency synchronous zone governed the translational velocity of SMRs, whereas the orientation of the precession axis determined the direction control, demonstrating the capability of SMRs for wireless navigation toward the targeted macrophage along a preset path at a set pace. Interestingly, SMR performed an in situ rotational motion around the targeted macrophage in an *x*–*y* rotational magnetic field. Mechanical signals generated by the rotational motion of SMRs induced the polarization of macrophages from M0 to M2 phenotype, which was attributed to Piezo1 and its downstream activating protein‐1 (AP‐1)‐CCL2 pathways.

## 2 RESULTS AND DISCUSSION

### 2.1 Fabrication and characterization of hollow iron oxide nanoparticles (HIONPs)

**FIGURE 2:**
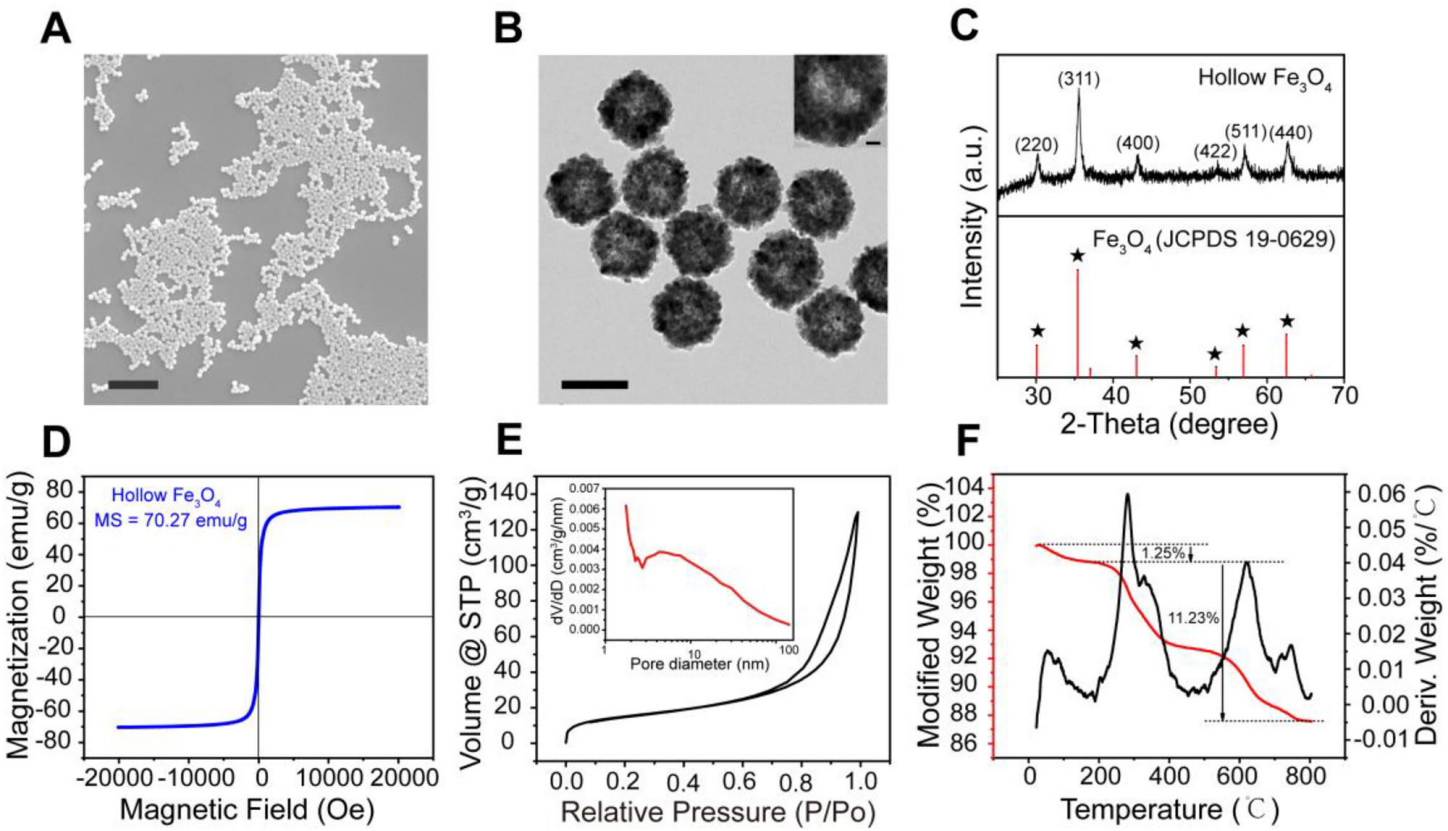
Characterizations of HIONPs. (A) SEM image of HIONPs, scale bar was 5 μm. (B) TEM image and enlarged view (inset) of HIONPs, scale bars were 200 and 20 nm, respectively. (C) XRD patterns of HIONPs. (D) Magnetic hysteresis loops of HIONPs at room temperature. (E) N_2_ adsorption/desorption isotherms and pore‐size distribution (inset) of HIONPs. (F) TGA‐DTG curves of HIONPs.

HIONPs with polymer polyacrylamide (PAM) modification were first synthesized through a well‐established hydrothermal approach.^[^ ^39^ ^]^ Scanning electron microscopy (SEM) (Figure 2A) and transmission electron microscope (TEM) images (Figure 2B) showed that the prepared nanoparticles were uniformly hollow nanospheres with a diameter of around 225 nm, and dynamic light scattering further determined the hydrodynamic dimensions (Figure S1). It could be seen that HIONPs were clusters of nanocrystals, which were smaller than the superparamagnetic critical size of Fe_3_O_4_ (Dp = 30 nm),^[^ ^40^ ^]^ indicating that the obtained HIONPs were superparamagnetic. Powder X‐ray diffraction (XRD) measurement demonstrated that the diffraction peaks of HIONPs closely resembled magnetite (Figure 2C). X‐ray photoelectron spectroscopy (XPS) spectra were further used to confirm the successful synthesis of magnetite. The Fe 2p XPS spectra (lower inset in Figure S2) illustrated the peak positions of Fe 2p1/2 and Fe 2p3/2 appeared at 710.3 and 724.0 eV respectively, which were consistent with the XPS data for Fe_3_O_4_.^[^ ^41^, ^42^ ^]^ At the same time, the XPS pattern of O 1s region (upper inset in Figure S2) showed a peak at 529.8 eV that corresponded to O‐Fe in magnetite phase,^[^ ^43^ ^]^ further proving that our simple was magnetite rather than maghemite. The magnetic property of HIONPs was then measured using a superconducting quantum interference device, and HIONPs had a saturation magnetization value of 70.27 emu g^−1^. The hysteresis loop showed reversible characteristics with no hysteresis in Figure 2D, which further confirmed the superparamagnetic property of our HIONPs. This meant that HIONPs could be easily magnetized (Figure S3) and manipulated by an external magnetic field, and lose magnetization without remaining when the magnetic field was removed. N_2_ adsorption and desorption analysis of HIONPs demonstrated that the Brunauer–Emmett–Teller surface area was 55.51 m^2^ g^−1^. The N_2_ adsorption‐desorption isotherm (Figure 2E) was classified as type IV,^[^ ^44^ ^]^ and the pore size distribution obtained from non‐local density functional theory (NLDFT) calculation was 0.5–147.6 nm (Figure S4).^[^ ^45^, ^46^ ^]^ Furthermore, mesopores size distribution was calculated with the Barrett–Joyner–Halenda model using the adsorption isotherms, with an average pore size of 16.74 nm (inset in Figure 2E). The hollow mesoporous structure of HIONPs helps minimized sedimentation, ensuring that the microrobots formed by the self‐assembly of nanoparticles move in fluid rather than on the bottom. There were several stages of mass loss in the thermal gravimetric (TG) curves of HIONPs (Figure 2F). The initial weight loss (1.25%) at 200°C was probably because of the water removal, whereas the weight loss (11.23%) from 200°C to 800°C could be attributed to the polymer coating. This result confirmed the presence of a polymeric PAM layer on HIONPs, which imparted good colloidal stability to HIONPs.^[^ ^44^ ^]^ The colloidal stability was further proved by the zeta potential of −32.2 ± 0.53 mV (Figure S5).^[^ ^47^ ^]^

### 2.2 Self‐assembly of chain‐like self‐assembled micro‐robots (SMRs)

**FIGURE 3:**
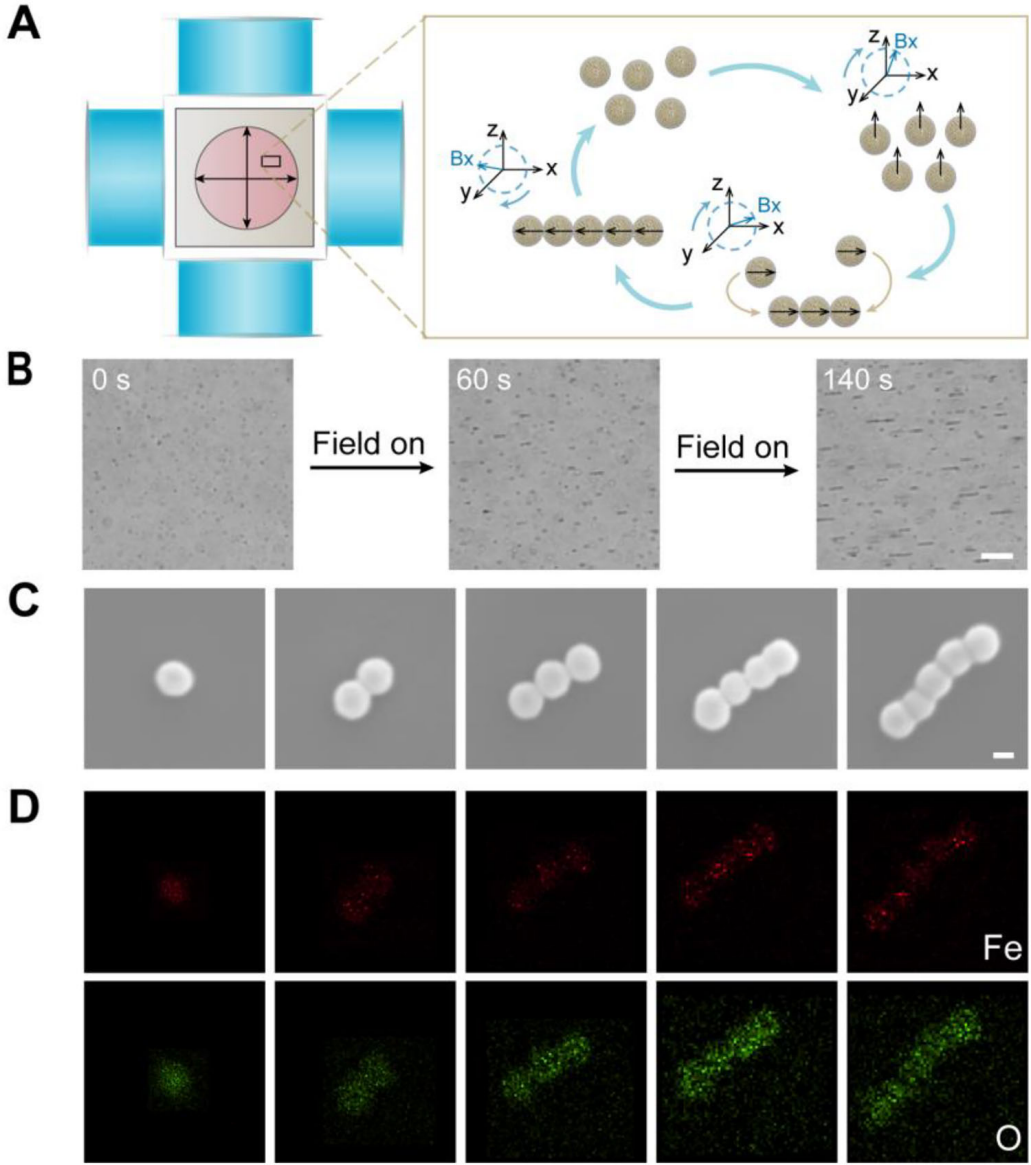
Self‐assembly and characterizations of chain‐like SMRs. (A) Schematic of chain‐formation process. (B) Microscope images during self‐assembly of HIONPs under magnetic field, scale bars were 10 μm. (C) SEM images and (D) EDS elemental mappings of SMRs with different chain lengths, scale bars were 200 nm.

After confirming the successful fabrication of HIONPs, HIONPs were added into cell culture dish under RMF for the formation of SMRs. Here, customized five‐coil magnetic field generator was used as a magnetic control system, which consisted of two pairs of orthogonal electromagnetic coils and a solenoid. Coordinate *o*‐*xyz* was established along the axis of the coil. To generate RMF, it is necessary to have at least two pairs of electromagnetic coils, which have a sinusoidal output with a 90° phase delay. Under a RMF in the *x*–*z* plane, HIONPs were subjected to dipole–dipole interactions (both repulsive and attractive) that varied periodically over time.^[^ ^48^ ^]^ When the direction of magnetic field was perpendicular to the connecting line of HIONPs, the particles were repelled and separated by dipole‐dipole repulsion, while the particles approached each other due to dipole‐dipole attraction as the angle between the direction of magnetic field and the connecting line was zero.^[^ ^37^ ^]^ After several cycles, the short chain was continuously extended and ultimately formed parallel chain microrobots along the direction of magnetic field (Figure 3A). It should be noted that, under the RMF, the chain‐like SMRs formed by monodispersed HIONPs would go through three processes: Brownian motion, dynamic self‐assembly, and deformation‐based motion. Once forming the chain‐like SMRs, the chains would be deformed into chiral structures by magnetic and hydrodynamic forces to generate propulsion (Movie S1). As shown in Figure 3B, the chain length increased with the acting time of magnetic field, which was also affected by magnetic field intensity and frequency, fluid viscosity, and field direction as reported.^[^ ^34^ ^]^ Besides, we found that the assembly efficiency was improved with the increased concentration of nanoparticles. In the case of low concentration, nanoparticles had a low chance to unite after the collision due to the weak dipole–dipole interactions at a long distance. Chain‐like SMRs were composed of HIONPs connected one by one as shown in SEM images (Figure 3C), and the elemental analysis by energy‐dispersive spectroscope (EDS) (Figure 3D) further demonstrated the existence of Fe and O elements.

### 2.3 Elastohydrodynamics of chain‐like SMRs at low Reynolds number

After confirming the structure and magnetic assembly of HIONPs, motion behavior of chain‐like SMRs was then investigated. The prerequisite for the propulsion of chain‐like SMRs was chiral deformation caused by the rotary drive and flexible structure at low Reynolds number as reported.^[^ ^35^, ^49^, ^50^ ^]^ Self‐assembled chains based on superparamagnetic nanoparticles under RMF were normally flexible.^[^ ^51^ ^]^ Flexible microswimmers based on the self‐assembly of magnetic nanoparticles would stretch and bend under hydrodynamic and magnetic forces, resulting in deformation, as previously reported.^[^ ^35^ ^]^ However, the principle of chiral deformation of chain‐like SMRs and the specific deformation structure need to be investigated further, because in the previous report, Hooke's law was only used to simulate the stretching and bending free energies and briefly mentioned that the dipole interaction between particles contributed to the deformation.^[^ ^35^ ^]^ The hydrodynamics of S shaped elastic deformation of paramagnetic particle chains under RMF has been studied.^[^ ^52^ ^]^ Combined with the working principle of chiral deformation of flexible nanowire motor, the behavior of the SMRs in the fluid under force was mathematically modeled. Chain‐like SMRs experienced elastic deformation to achieve stable chain shape under the action of magnetic force and viscous resistance along the chain, since inertia forces were negligible at low Reynolds number.^[^ ^53^ ^]^ We investigated the force of SMRs made up of *N* (*N* ≥ 3) nanoparticles under RMF in *x*–*z* plane.

HIONPs were magnetized with an induced dipole moment when RMF was applied based on the following equation:

(1) $$\mu =\frac{4}{3}\pi a^{3}\;\mu_{0}\chi H_{0},$$

where *a* is the radius of the particle, *μ*_0_ is the permeability of vacuum and *χ* is the particle susceptibility.

Radial component of dipole interaction force between *N* magnetic nanoparticles in uniform magnetic field is as follows:

(2) $$\vec{F_{m}}=\frac{3\mu^{2}}{4\pi \mu_{0}a^{4}}\;\sum_{i=-N/2}^{i=N/2}\left(3cos^{2}\alpha_{i}-1\right)\hat{r},$$

where *r* is the distance between the center of the sphere, and *α* is the angle between external magnetic field and the center of the sphere.

The rod‐shaped model was used to approximate the viscous resistance of SMRs:

(3) $$\vec{F_{d}}=\zeta_{⊥}\vec{v_{i}},$$

where $\zeta_{⊥}=\frac{4\pi \eta aN}{ln(N/2)}$ is the normal drag coefficient and $\vec{v_{i}}=\omega r_{i}\hat{r}$ is the velocity of the *i*th particle.

When chain‐like SMR was deformed, elastic bending force would be generated to minimize the bending energy. The elastic bending force was then calculated by taking the derivative of the bending energy $U_{bend}=\frac{1}{2}\int_{0}^{L}A{\left(\frac{d^{2}y}{dx^{2}}\right)}^{2}$, where *A* is the flexural rigidity. The elastic bending force is as follows:

(4) $$f=A\frac{\left(d^{4}y\right)}{dx^{4}}.$$

The elastic bending force increased linearly along the length of the chain,^[^ ^52^ ^]^ which made the end of the chain align with the field faster than other parts of the chain body, thus forming an “S‐shape.”^[^ ^52^, ^53^, ^54^ ^]^ When an RMF was applied in the *x*–*z* plane, the two ends of the chain would be in different stress environments because one end was closer to the solid surface and the other to the upper liquid level. As a result, “S‐shape” deformations in the *x*–*z* rotation plane may produce a spatially helical‐like structure, which is required for SMRs to move during rotation.

### 2.4 Magnetically actuated motion dynamics of SMRs

**FIGURE 4:**
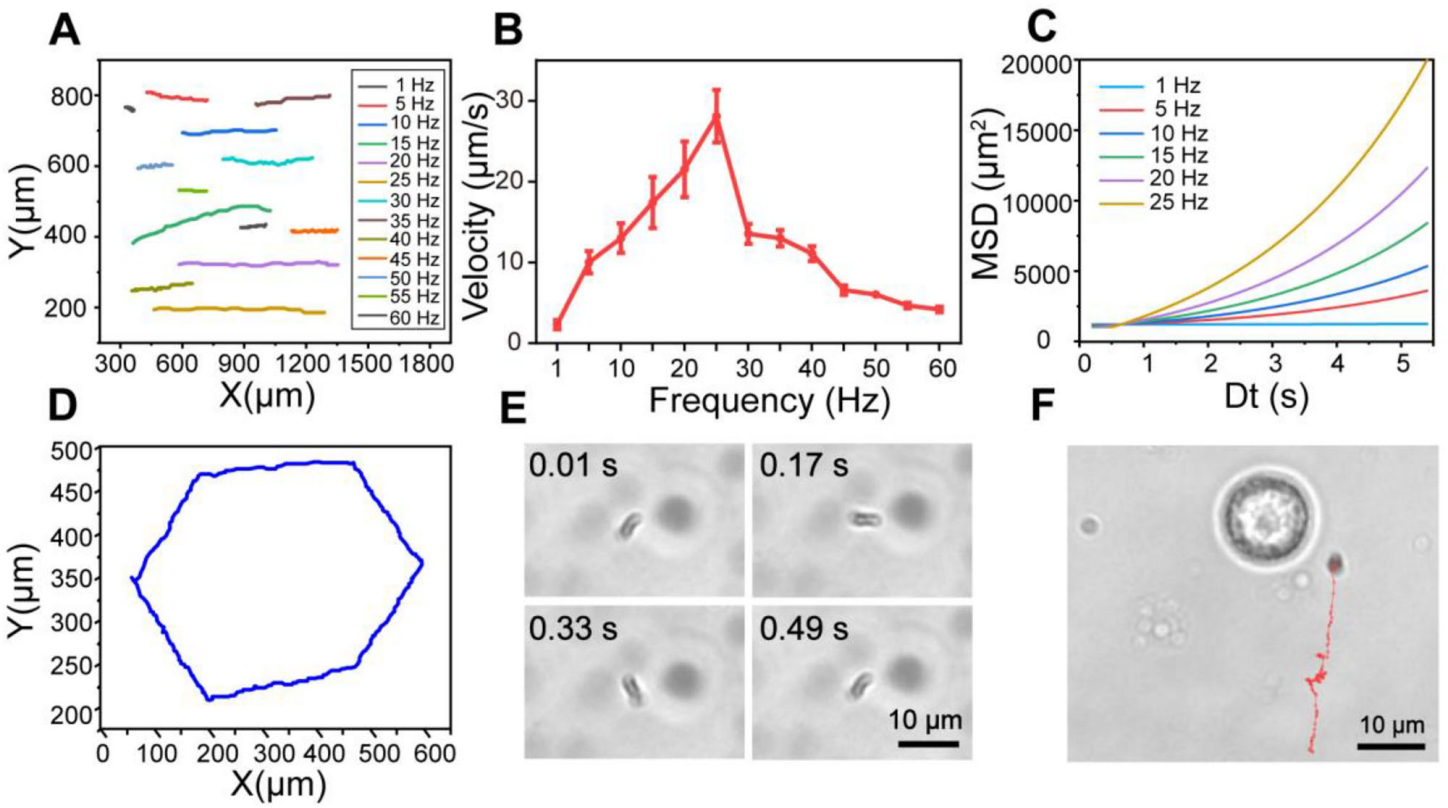
Motion behavior of SMRs. (A) Optical tracking trajectories of SMRs at different frequencies. (B) Velocity of SMRs at different frequencies in PBS. (C) MSD curves of SMRs at different frequencies. (*n* = 15). (D) Writing hexagonal trajectory by adjusting magnetic field. (E) Video snapshots of SMR rotation at different time intervals (1 Hz). (F) SMR approaching the targeted macrophage through magnetic navigation.

The chiral deformation of SMRs provides a necessary prerequisite for low Reynolds number swimming. We further investigated the motion behavior of SMRs under different magnetic frequencies using RMF in the *x*–*z* plane since the behavior of MMRs is closely related to applied field frequency.^[^ ^55^ ^]^ The trajectories at different frequencies (1, 5, 10, 15, 20, 25, 30, 35, 40, 45, 50, 55, and 60 Hz) were recorded by optical tracking (Figure 4A). The velocity of SMRs increased almost linearly to a peak (28.10 μm s^−1^) and subsequently decreased as the rotating frequency increased (Figure 4B), which is consistent with the motion behavior of spiral microrobots under RMF.^[^ ^33^ ^]^ The frequency at which the speed reached its maximum was known as the step‐out frequency in this case 25 Hz.^[^ ^56^ ^]^ When the driving frequency increased from 1 to 25 Hz, the velocity of SMRs increased from 2.307 to 28.10 μm s^−1^, whereas it decreased to 4.173 μm s^−1^ as the frequency increased to 60 Hz (Figure S6). The mean square displacement (MSD) under different magnetic field frequencies was calculated according to x‐y coordinates of SMRs trajectories (Figure 4C and Figure S7). The parabolic‐like function curve reflected the directional motion of SMRs and the slope represented the speed of motion,^[^ ^57^ ^]^ which was consistent with the above‐mentioned rule of velocity changing with frequency.

Interestingly, the motion behavior of SMRs was split into the synchronous and asynchronous regions with frequency since the phase lag angle between the long axis of SMRs and the RMF (Figure 4B).^[^ ^58^ ^]^ Due to the balance between magnetic torque and viscous torque under the operation of RMF, SMRs rotated passively and showed a delayed reaction, resulting in phase lag angle.^[^ ^54^ ^]^ At low frequencies, the phase lag angle was constant, SMRs rotated synchronously with the field, and the speed increased in a coordinated manner with deformation periods. The phase lag angle increased with time when the frequency was beyond the out‐of‐step one, and SMRs were no longer synchronized with magnetic field since the viscous torque exceeded the magnetic torque.^[^ ^53^ ^]^ Therefore, SMRs were subsequently slowed or reoriented to regain balance (Movie S2).^[^ ^59^ ^]^ It was worth noting that the out‐of‐step frequency for SMRs with various sizes may differ, resulting in a few SMRs moving at different speeds or drifting in the same field of vision. In our experiment, the motion law was derived from most SMRs with an average size around 9.45 μm (according to the Image J calculation). Furthermore, the phenomena of chains becoming shorter and even breaking was noticed in Movie S2, which could be attributed to the rising viscous resistance that tends to fragmentation.^[^ ^58^ ^]^ It has been reported that the average size of stable SMRs was related to Mason number, a dimensionless ratio between the magnetic force and the viscous resistance.^[^ ^60^ ^]^

Moreover, the motion direction of SMRs could also be controlled by adjusting the phase of coil axis (*x*/*y*/*z* axis) as the orientation of the precession axis changed.^[^ ^34^ ^]^ The homemade magnetic system with a phase control range of 0–360° enables precise control of SMRs to the target site. SMRs were manipulated to travel in a predetermined path (Movie S3), and the hexagonal trajectory was depicted in Figure 4D and Figure S8. Meanwhile, the rotational motion of SMRs in the *x*–*y* plane was also investigated, because mechanical signals generated by the rotational motion of rod‐like magnetic nanomotors have been used for surface‐enhanced Raman spectroscopy sensing and efficient enzymelinked immunosorbent assay detection.^[^ ^38^, ^61^ ^]^ It is worth mentioning that, unlike our system, these magnetic nanomotors were rigid rod‐like structures driven by a gradient magnetic field and switching to RMF for rotational motion. Superimposed experimental snapshots were used to depict the rotational motion of SMRs (Figure 4E). Interestingly, SMRs were able to maintain a fixed‐point rotation without translation over a period of time (Movie S4). It differed from the above self‐assembly video in which SMRs would start deformation‐based movement once they form a chain under the RMF. We hypothesized that it was because of the different forces acting on SMRs. When rotating in the *x*–*y* plane, both ends of the chain SMRs experienced the same force, which was different from that in the *x*–*z* plane due to the influences such as liquid layer thickness and bottom interface effect.^[^ ^34^ ^]^ Here, we chose fixed‐point rotation of SMRs in the *x*–*y* plane as the source of mechanical signals because it was advantageous for efficient stimulation of target cells. Figure 4F and Movie S5 showed an SMR approaching a targeted macrophage and then continuously rotating around the cell while switching to the *x*–*y* rotation plane.

As mentioned above, we developed a self‐assembly microrobot based on deformation. Other similar magnetically assembled microrobots have also been reported previously. Micro‐wheels and the linear chain‐like microrobots are self‐assembled by micron‐scale magnetic particles and share the same propulsion mechanism by symmetry breaking with nearby surfaces.^[^ ^34^, ^62^ ^]^ In addition, the rod‐like magnetic micromotors utilizing rotational motion mechanically improve detection efficiency, however, which are rigid chains that are driven by a gradient magnetic field and switching to RMF for actively rotating.^[^ ^38^, ^61^ ^]^ Our SMRs are based on nano‐scale magnetic particles which emphasize their own deformation drive and avoid the uncontrollable motion brought by surface at different angles, at the same time, integrate forward and rotational motion in one RMF, which offers great potential for biological application in various scenarios.

### 2.5 The M0 to M2 polarization capability and mechanism of SMRs

**FIGURE 5:**
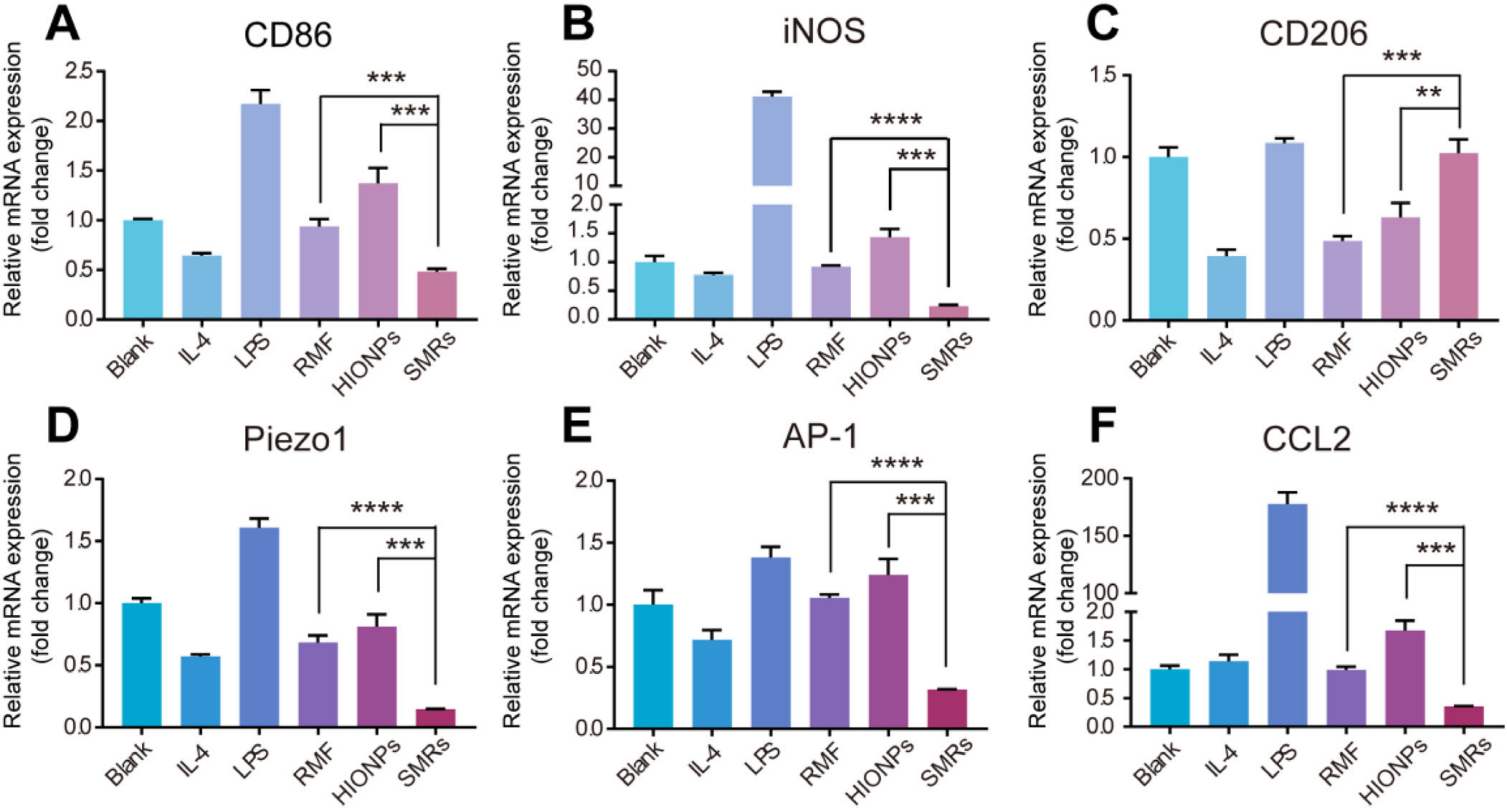
Effect and mechanism of SMRs on M0 macrophages polarization. (A–C) The relative gene expression of M1 and M2 macrophage markers after different treatments. (D–F) Signaling pathway studies of M0 to M2 macrophages induced by SMRs treatment. *n* = 3. (*p < 0.05, **p < 0.01, ***p < 0.001, and ****p < 0.0001, *t*‐test analysis).

The effect of SMRs as local mechanical signal generators on M0 macrophages polarization was then studied. We first evaluated the biocompatibility of HIONPs and SMRs (Figures S9 and S10). The biocompatibility of SMRs was tested by using HIONPs, which were treated with an RMF for 20 min and then removed and further incubated for 24 h. After being incubated with various concentrations of HIONPs/SMRs for 24 h, RAW 264.7 cells still maintained relatively high cell viability.^[^ ^63^ ^]^ Moreover, the results showed an apparent significant increase in viability at low concentrations of nanoparticles, which is consistent with the previous report that superparamagnetic iron oxide nanoparticles promote the growth of RAW264.7 cells.^[^ ^64^ ^]^ The cell viability results for SMRs showed a similar trend. Then 100 μg mL^−1^ HIONPs were added into a culture dish seeded with RAW 264.7 macrophages, followed by RMF of *x*–*y* plane treatment for 20 min. Under RMF, chain‐like SMRs were constructed and performed rotational motion in the macrophage microenvironment. Only HIONPs or RMF treatment was used as conditional controls to understand the impact of nanoparticles and magnetic field on macrophage polarization. Lipopolysaccharide (LPS, 1 μg mL^−1^)/interleukin‐4 (IL‐4, 20 ng mL^−1^) stimulation was used as positive/negative control.^[^ ^65^ ^]^ After treatments, the levels of macrophage polarization were investigated by quantifying the expression of three classic M1 markers (CD86, iNOS, and TNF‐α) (Figure 5A,B and Figure S11) and a classic M2 markers (CD206) (Figure 5C). Compared with blank/IL‐4 treatment, RAW 264.7 macrophages after LPS treatment displayed high expression of iNOS (Figure 5B) and inflammatory cytokine (TNF‐α) (Figure S11) with 41.32/418.01 folds change respectively, indicating that the M0 macrophages used in our experiments had a good response to polarized stimulation signals. While for our strategy, SMRs‐treated M0 macrophages showed significantly decreased expression levels of M1 classic markers, which was demonstrated from both protein (CD86, iNOS) and cytokine (TNF‐α) perspectives. Moreover, the M2 classic markers (CD206) were significantly upregulated by our SMRs, suggesting that the mechanical signal generated by the rotation of SMRs polarized M0 macrophages toward M2 phenotype.

To understand the possible mechanism for M2 polarization, the polarization signaling pathway related to mechanical responses was further explored. It was reported that Piezo1 ion channel normally responded to cyclical hydrostatic pressure and triggered the activation of AP‐1, inducing significant up‐regulation of pro‐inflammatory genes.^[^ ^66^ ^]^ For classical M1 signaling pathway, the downstream of AP‐1 is CCL2. ^[^ ^65^ ^]^ Therefore, the expression levels of Piezo1, AP‐1, and CCL2 genes in SMRs‐treated M0 macrophages were detected by qPCR. As shown in Figure 5D–F, the macrophages treated with SMRs under magnetic field significantly down‐regulated the gene expression of Piezo1, AP‐1, and CCL2 when compared to the cells treated with only HIONPs or RMF, thus demonstrating that the mechanical signal generated by extracellular rotation of SMRs drove M2 polarization via inhibiting Piezo1‐AP‐1‐CCL2 signal pathway.

We demonstrated that SMRs successfully regulated macrophages into anti‐inflammatory phenotypes via the mechanically responding ion channel Piezo1. It has been a major problem in the field of micro/nano‐robots that tailoring biomedical applications for autonomously moving micro/nano‐robots. The biggest characteristic of micro‐nano robot is the autonomous movement, in addition to which is the mechanical signal generated by the movement. As reported previously, the mechanical stimulation of MNRs has been used to improve the detection efficiency,^[^ ^38^ ^]^ promote the penetration of cell membranes and conduct single‐cell surgery.^[^ ^67^, ^68^, ^69^ ^]^ Our system is committed to utilize mechanical stimulation for cell fate regulation, opening a new avenue for the adaptive biological applications of MNRs. Moreover, our strategy provides a new mechanical signalsource beyond the existing implanted materials and external mechanical loading devices.

## 3 CONCLUSION

In summary, magnetically driven SMRs self‐assembled by HIONPs were successfully designed and fabricated as a novel mechanical signal generator for M0 to anti‐inflammatory associated M2 macrophage polarization. We not only provide a theoretical and experimental demonstration of magneto‐driven principles and motion behavior of chain microrobots, but also construct a simple, effective, and stable mechanical signal generator for macrophage polarization. Under a RMF in the *x*–*z* plane, SMRs exhibit elastic deformation in response to magnetic force and viscous resistance according to mathematical model, gaining “S‐shape” chiral structure to enable forward propulsion. The motion behavior of the SMRs was similar to that of the helical micro‐robots, including frequency‐related motion behavior and precisely controllable navigation, which further explained the chiral deformation‐based propulsion mechanism. By applying x‐y RMF, the SMRs showed fixed‐point rotational motion, acted as mechanical signal generator that successfully induced M2 macrophage polarization by blocking the Piezo1‐ AP‐1‐CCL2 signaling pathway. Our strategy represents an effective, stable, and non‐invasive mechanical approach to achieve microenvironmental immune cell fate regulation. We envision that our platform has great potential to provide exciting opportunities in a wide range of cell fate regulation and subsequent disease theranostics since hollow magnetic particles may be further functionalized as carriers/magnetic resonance probes. In addition, by combining with the mechanical signal sensing device and other technical, SMRs would revolutionize many other conventional mechanical signal loading strategies.

## 4 EXPERIMENTAL SECTION

### 4.1 Synthesis of HIONPs

HIONPs were synthesized by solvothermal method based on previous reports with slight modifications.^[^ ^39^, ^44^ ^]^ Briefly, 2 mmoL ferric chloride hexahydrate (FeCl_3_•6H_2_O), 4 mmoL trisodium citrate dihydrate (C_6_H_5_O_7_Na_3_•2H_2_O) and 6 mmoL urea (CH_4_N_2_O) were first dissolved in 40 mL distilled water. Then, 0.3 g of PAM powder was slowly added under continuous stirring until it was completely dissolved. The resulting mixture was then transferred and sealed in a Teflon‐lined stainless‐steel autoclave (50 mL capacity) at 200°C and maintained for 12 h. After cooling to room temperature, the black product was collected by centrifugation and washed alternately with ethanol and deionized water several times. Then, the obtained HIONPs were finally stored at 4°C for further use.

### 4.2 Cytotoxicity assay

3‐(4,5‐Dimethylthiazol‐2‐yl)−2,5‐diphenyltetrazolium bromide (MTT) assay was used to determine the cytotoxicity of HIONPs. RAW 264.7 macrophages were seeded (1 × 10^4^ cells per well) into 96‐well plates and cultured for 24 h. The media were replaced with fresh ones containing different concentrations of HIONPs and further incubated for 24 h. The media were then removed and MTT solution (0.5 mg mL^−1^ in medium) was added for another 4 h incubation. Subsequently, the media were replaced by DMSO (150 μL per well) and measured by a microplate reader (Biotek 800 TS, USA) at absorbance of 490 nm.

### 4.3 Quantitative PCR (qPCR) analysis

RAW 264.7 macrophages were seeded (3 × 10^5^ cells per well) into confocal dishes (20 mm) and incubated for 24 h. The media were then replaced with fresh ones containing HIONPs (100 μg mL^−1^) and the resulting cells were then treated with or without RMF for 20 min (2.3 mT, 25 Hz). There were six groups. Under RMF, HIONPs self‐assembled and formed micro‐robots (SMRs) as experimental group, which was used to investigate the effect of mechanical signals generated by the motion of SMRs on macrophages within 20 min. Only HIONPs or RMF treatment was used as a condition control to understand the effects of nanoparticles and magnetic fields on macrophage polarization. The untreated group with basal medium was used as blank control. Positive/negative control groups were temporarily supplemented with basal medium in this step. Subsequently, HIONPs were washed off with PBS (0.1 m, pH = 7.4) to avoid the particles' influence on the macrophages. The cells were then cultured for additional 6 h with fresh medium, LPS (1 μg mL^−1^), and IL‐4 (20 ng mL^−1^). LPS and IL‐4 stimulation were used as positive/negative controls. mRNA expression levels of CD86, iNOS, CD206, Piezo1, AP‐1, CCL2, TNF‐α were evaluated by qPCR analysis based on the following procedures. The cells were harvested and RNA was extracted using Cell Total RNA Isolation Kit (FOREGENE, China). Total RNA (1 μg) was then reverse‐transcribed into cDNA with cDNA Reverse Transcription Kits (NovaBio). qPCR was carried out on a LC480 Real‐Time PCR detection system by using an SYBR Green PCR Master Mix kit (NovaBio). Circulating conditions were subject to manufacturer's protocol. The primers tested were listed in Table S1.^[^ ^65^, ^70^ ^]^ The relative expression of mRNA was quantified using 2^–ΔΔCt^ method.

## CONFLICT OF INTEREST STATEMENT

The authors declare no conflicts of interest.

## Supporting information

Movie S1. Self‐assembly of SMRs. (AVI)

Movie S2. Locomotion of SMRs under different rotation frequencies in PBS. (AVI)

Movie S3. SMR followed a specified hexagon trajectory. (AVI)

Movie S4. Rotation of SMRs under magnetic field with 2 Hz. (AVI)

Movie S5. SMR steered to the target cell and performed fixed‐point rotation under 2 Hz rotary magnetic field. (AVI)

FIGURE S1. Size distribution of HIONPs.
FIGURE S2. The XPS spectrum of the as‐prepared HIONPs and the high resolution XPS spectrum of Fe 2p (lower inset) and O 1s (upper inset) from the fractured surface of the HIONPs.
FIGURE S3. Photographs of HIONPs treated by a magnet (∼ 260 Gs).
FIGURE S4. NLDFT pore size distribution of HIONPs.
FIGURE S5. Zeta potential of HIONPs.
FIGURE S6. The tracking trajectories of SMRs under magnetic field with different frequencies (1, 25, and 60 Hz).
FIGURE S7. MSD curves of SMRs under different frequencies (> 25 Hz).
FIGURE S8. Video snapshots of hexagon trajectory written by SMR.
FIGURE S9. Relative cell viabilities of RAW 264.7 macrophages after treating with different concentrations of HIONPs.
FIGURE S10. Relative cell viabilities of RAW 264.7 macrophages after treating with different concentrations of SMRs.
FIGURE S11. Gene expression level of TNF‐α after treating with SMRs. Statistical significance was determined using unpaired *t*‐test. ** *p* < 0.01, **** *p* < 0.0001. vs. control (RMF or HIONPs group).
TABLE S1. Primer sequences for qPCR analysis.

## Data Availability

All data of this work are present in the article and Supporting Information. The other data that support the findings of this work are available from the corresponding author upon reasonable request.
