# Supplementary material for: Mobile mechanical signal generator for macrophage polarization
Source: Exploration (Beijing). 2023 Apr 3;3(2):20220147. doi: 10.1002/EXP.20220147 (PMC10190931; doi:10.1002/EXP.20220147)
Supplement: Supplementary file 6 — FIGURE S1. Size distribution of HIONPs. FIGURE S2. The XPS spectrum of the as‐prepared HIONPs and the high resolution XPS spectrum of Fe 2p (lower inset) and O 1s (upper inset) from the fractured surface of the HIONPs. FIGURE S3. Photographs of HIONPs treated by a magnet (∼ 260 Gs). FIGURE S4. NLDFT pore size distribution of HIONPs. FIGURE S5. Zeta potential of HIONPs. FIGURE S6. The tracking trajectories of SMRs under magnetic field with different frequencies (1, 25, and 60 Hz). FIGURE S7. MSD curves of SMRs under different frequencies (> 25 Hz). FIGURE S8. Video snapshots of hexagon trajectory written by SMR. FIGURE S9. Relative cell viabilities of RAW 264.7 macrophages after treating with different concentrations of HIONPs. FIGURE S10. Relative cell viabilities of RAW 264.7 macrophages after treating with different concentrations of SMRs. FIGURE S11. Gene expression level of TNF‐α after treating with SMRs. Statistical significance was determined using unpaired t‐test. ** p < 0.01, **** p < 0.0001. vs. control (RMF or HIONPs group). TABLE S1. Primer sequences for qPCR analysis. [file EXP2-3-20220147-s004.docx]

Supporting Information

Mobile Mechanical Signal Generator for Macrophage Polarization

*Jiamiao Jiang,^‡^ Fei Wang^,‡^ Weichang Huang,^‡^ Jia Sun, Yicheng Ye, Juanfeng Ou, Meihuan Liu, Junbin Gao, Shuanghu Wang, Dongmei Fu, Bin Chen, Lu Liu, Fei Peng* and Yingfeng Tu**

**1. Materials**

Ferric chloride hexahydrate (FeCl_3_•6H_2_O) was purchased from Aladdin. Trisodium citrate dehydrate (C_6_H_5_O_7_Na_3_•2H_2_O) was purchased from Alfa Aesar. Polyacrylamide (PAM) was obtained from Macklin. Lipopolysaccharide (LPS) and interleukin-4 (IL-4) were bought from Sangon Biotech (Shanghai, China). Unless otherwise specified, the chemicals used in this work were all purchased from Sigma-Aldrich. All the reagents were used as-received without further purification. All reagents for cell culture were bought from Gibco. The purified deionized water was prepared by the Milli-Q plus system (Millipore, USA).

**2. Cell culture**

RAW 264.7 macrophages were purchased from the Institute of Biochemistry and Cell Biology of the Chinese Academy of Sciences (Shanghai, China). RAW 264.7 macrophages were cultured in DMEM supplemented with 10% FBS and 1% PS at 37 °C with 5% CO_2_.

**3.** **Characterizations**

The hydrodynamic diameter and zeta potential of HIONPs were measured by dynamic light scattering (DLS, Malvern, Nano series ZSE, UK). The morphology of HIONPs was obtained through transmission electron microscopy (TEM) using a Tecnai G2 20 microscope operated at 200 kV. Scanning electron microscopy (SEM) and energy-dispersive X-ray spectroscopy (EDS) were recorded on a Phenom emission scanning electron microscope (Phenom, ProX, Netherlands). The phase structure of the samples was identified by Powder X-ray diffraction (XRD) on a D8 ADVANCE X-ray diffractometer. X-ray photoemission spectroscopy (XPS) was recorded on Thermo Fisher Nexsa. The magnetic properties of HIONPs were analyzed by a MPMS (SQUID) XL magnetic measuring system at 300 K in the applied maximum magnetic field of 10 kOe. N_2_ adsorption/desorption isotherms and pore size distributions of HIONPs were obtained by ASAP 2460 Surface Area and Porosity Analyzer. Specific surface area was calculated according to the Brunauer-Emmet-Teller (BET) model. The pore size distribution (including micropores and mesopores) was calculated by Non-Local Density Functional Theory (NLDFT) and the pore size distribution of mesopores was calculated using the adsorption branches of the nitrogen isotherms via the Barrett–Joyner–Halenda (BJH) model. Thermogravimetric analysis and derivative thermogravimetry (TGA-DTG) data were recorded with a thermal analysis instrument (SDT Q600, TA Instruments) with a heating rate of 10 °C min^−1^ in a Nitrogen gas flow of 100 mL min^−1^. The Fe contents of HIONPs was quantified by inductively coupled plasma mass spectrometry (ICP-MS, NexION 2000B, PerkinElmer).

**4. Magnetic control system**

The home-made magnetic manipulation system was composed of a five-coils magnetic field generator, function generator (FeelElec FY8300S-60M) and power amplifier (YINGPU MAGNETOELECTRIC, HEAS-20). The sine wave signal function obtained from the function generator was amplified by a power amplifier to drive the magnetic field generator to produce a rotating magnetic field.^[1]^

**5. Optical recording**

The samples were placed in a 20 mm confocal dish filled with PBS, and the motion were observed with an inverted optical microscope (Nikon, Ti-2, Japan) with a 40X objective lens. Video was recorded by a sCMOS camera (PCO, pco.edge 4.2, Germany).

**Supplementary Figures**

**
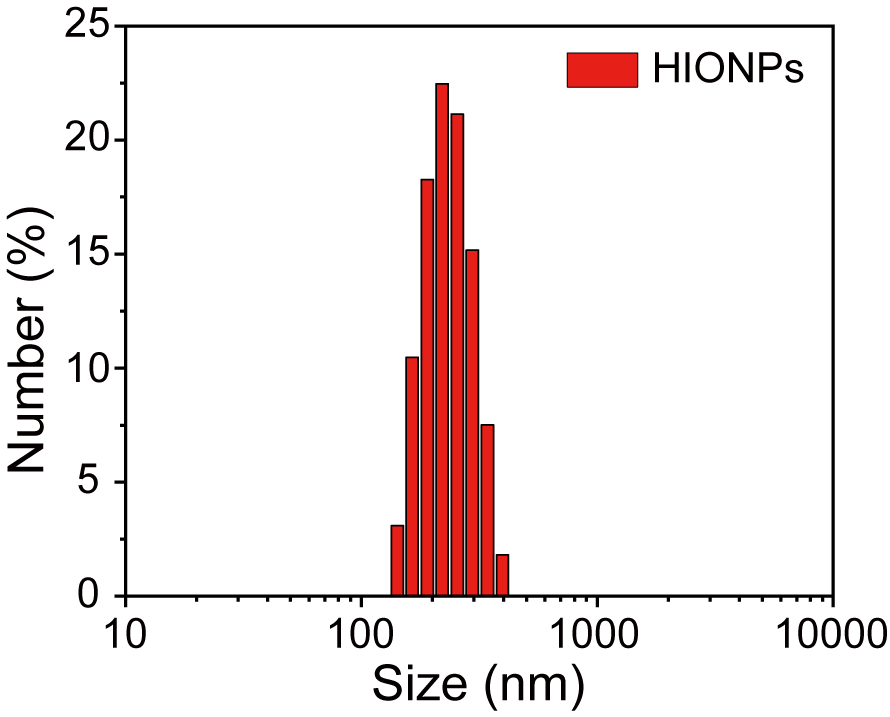
**

**FIGURE S1.** Size distribution of HIONPs.


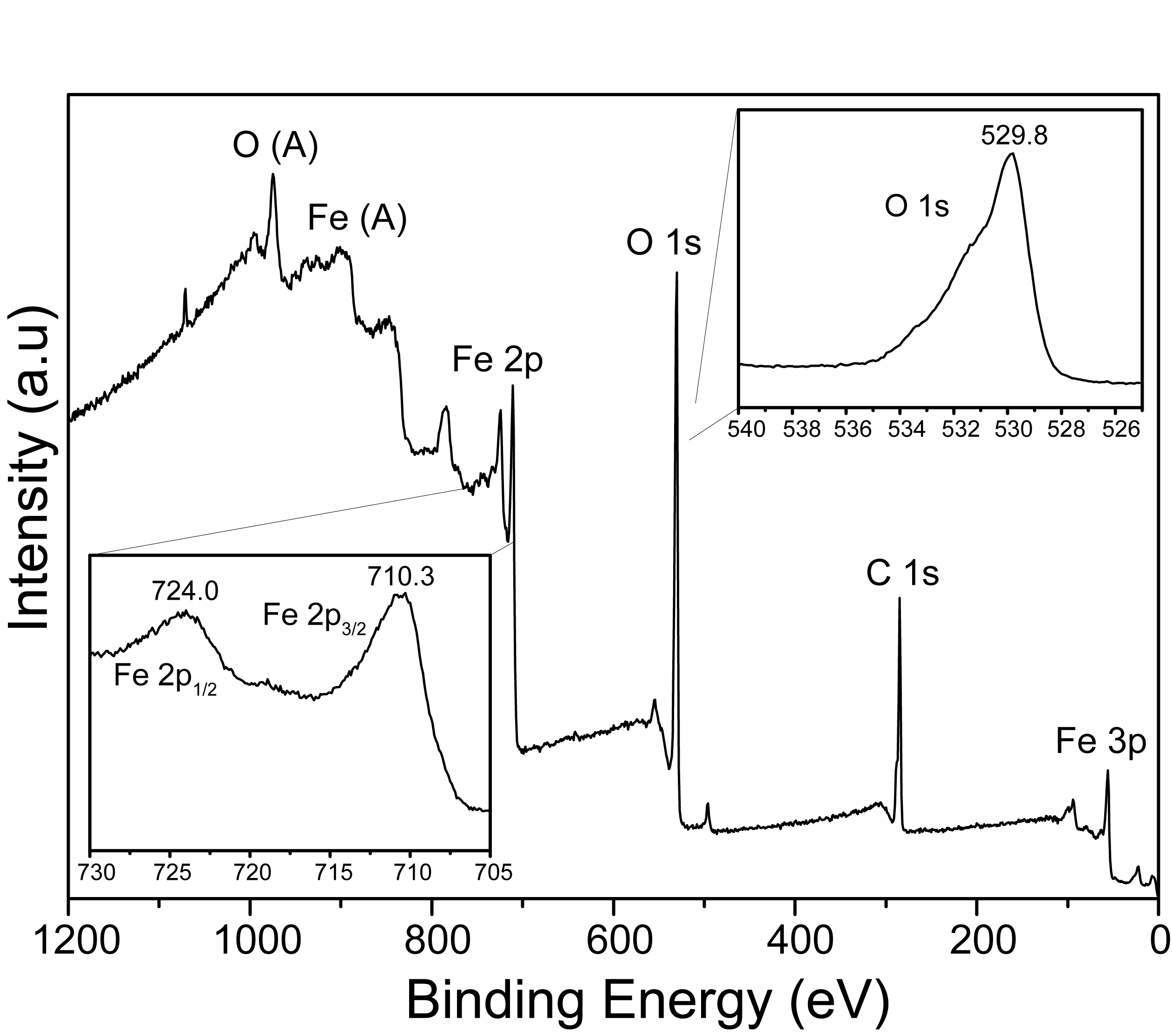


**FIGURE S2.** The XPS spectrum of the as-prepared HIONPs and the high-resolution XPS spectrum of Fe 2p (lower inset) and O 1s (upper inset) from the fractured surface of the HIONPs.


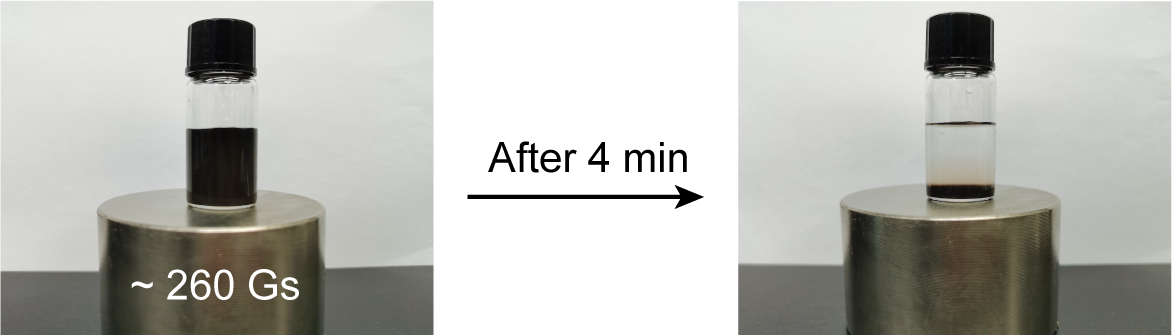


**FIGURE S3.** Photographs of HIONPs treated by a magnet (~260Gs).


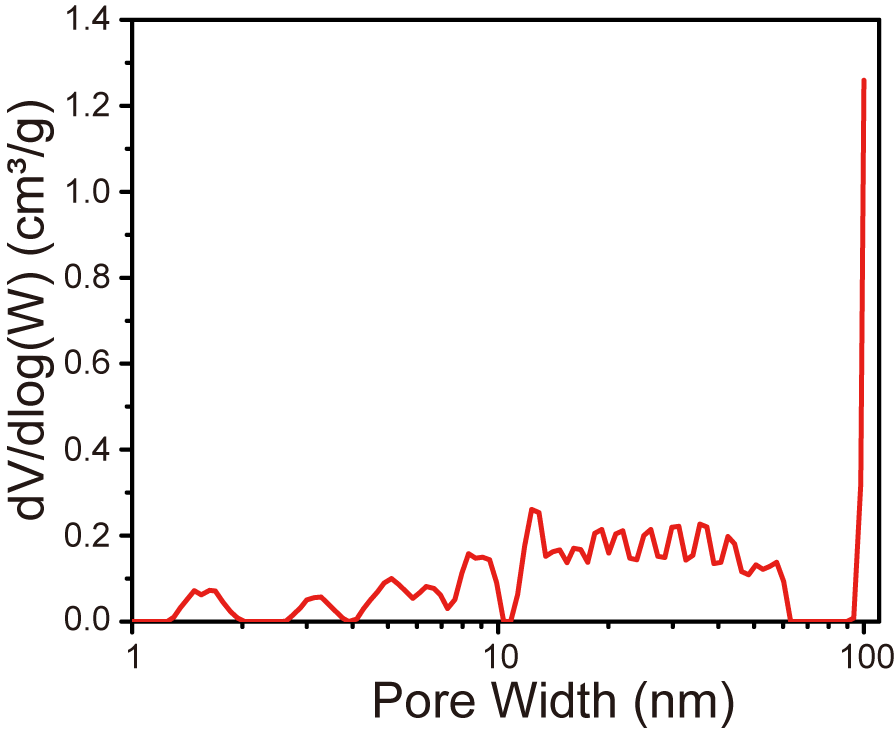


**FIGURE S4.** NLDFT pore size distribution of HIONPs.


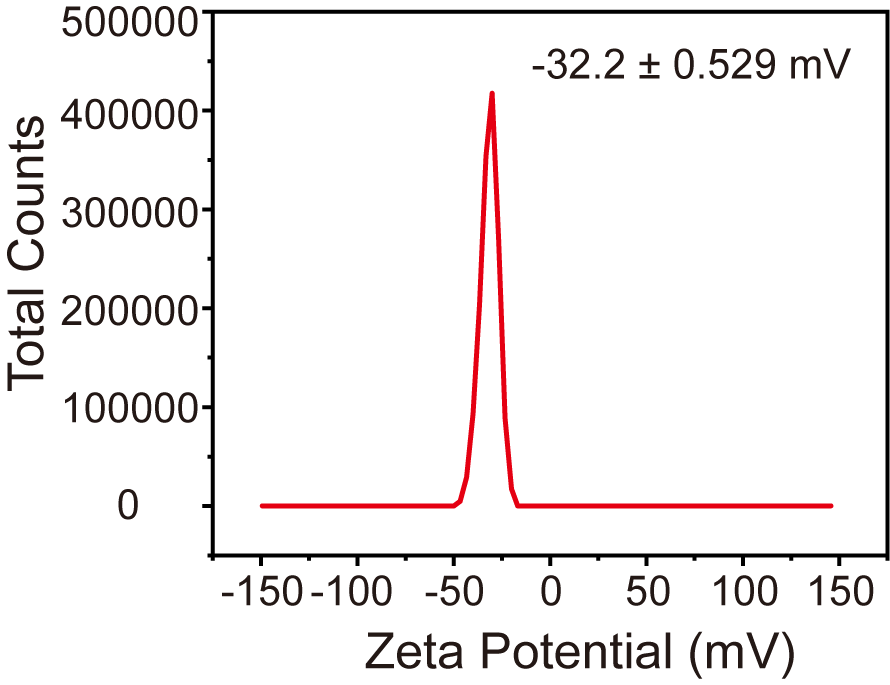


**FIGURE S5.** Zeta potential of HIONPs.


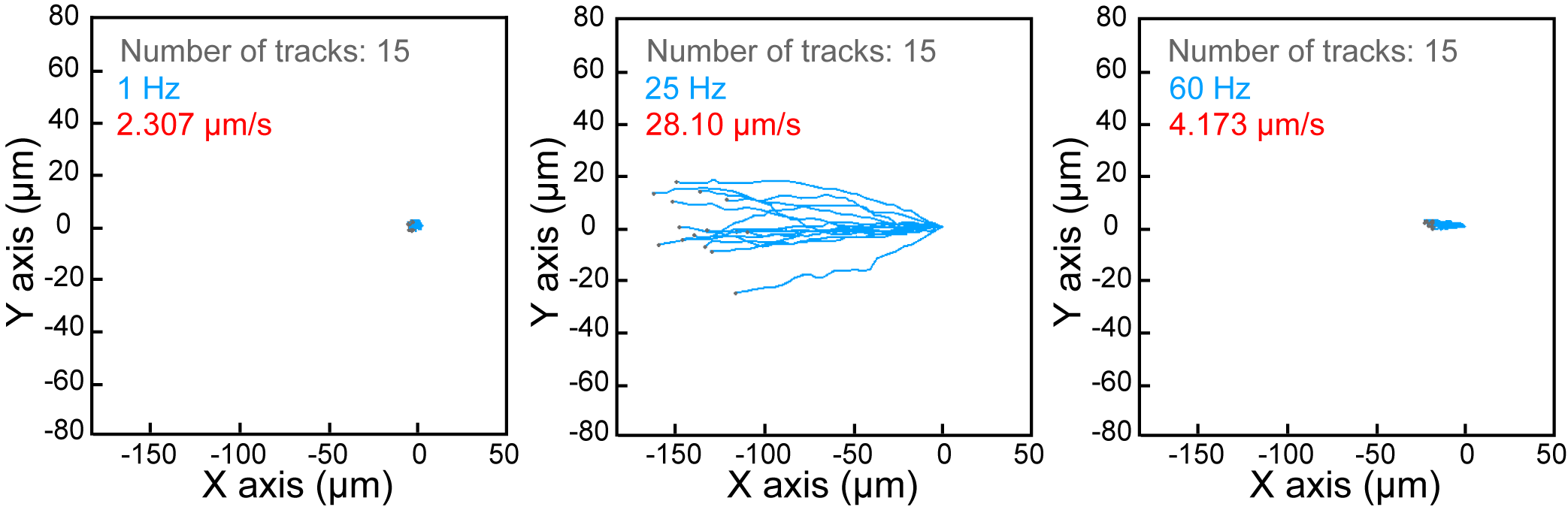


**FIGURE S6.** The tracking trajectories of SMRs under magnetic field with different frequencies (1 Hz, 25 Hz and 60 Hz).


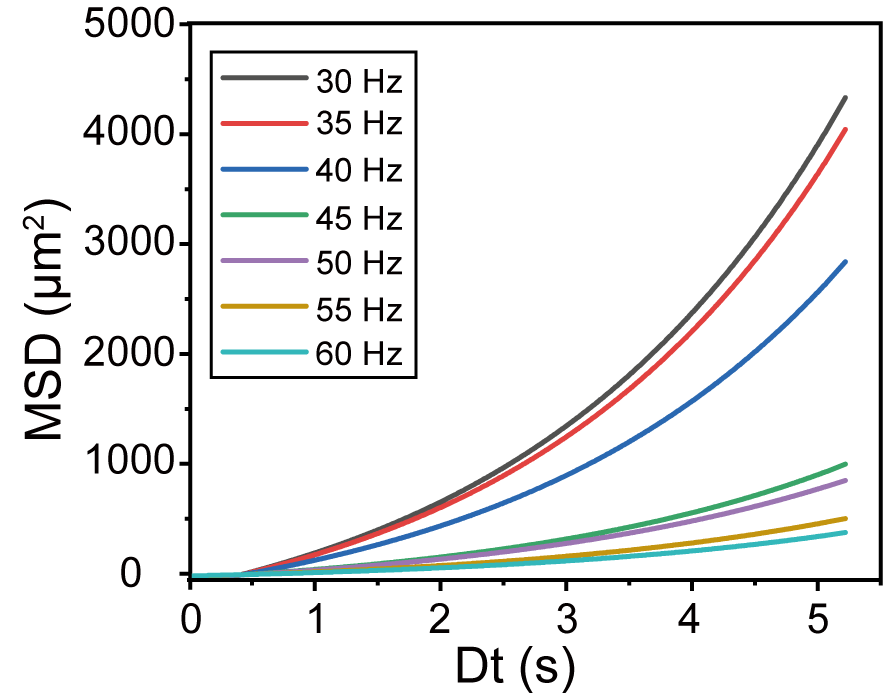


**FIGURE S7.** MSD curves of SMRs under different frequencies (>25 Hz).


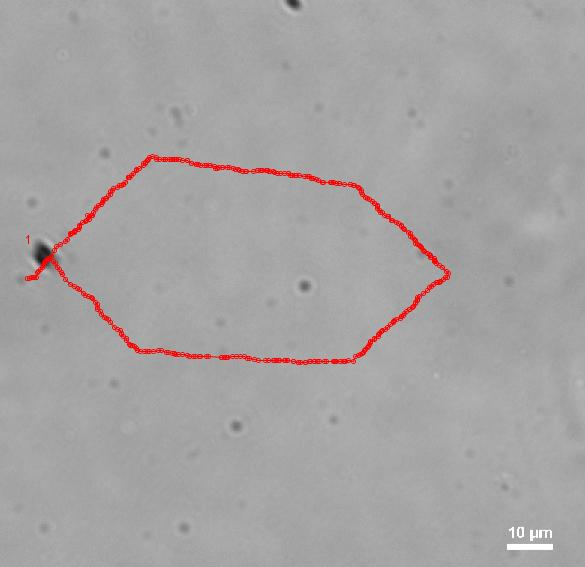


**FIGURE S8.** Video snapshots of hexagon trajectory written by SMR.


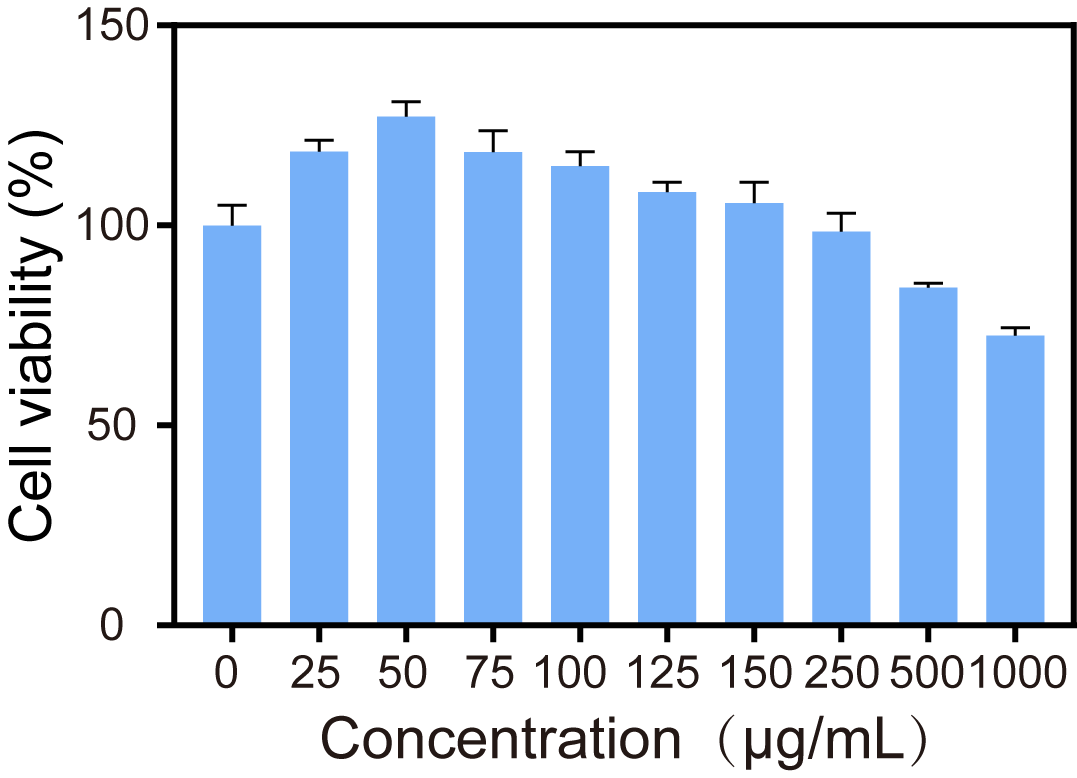


**FIGURE S9.** Relative cell viabilities of RAW 264.7 macrophages after treating with different concentrations of HIONPs.


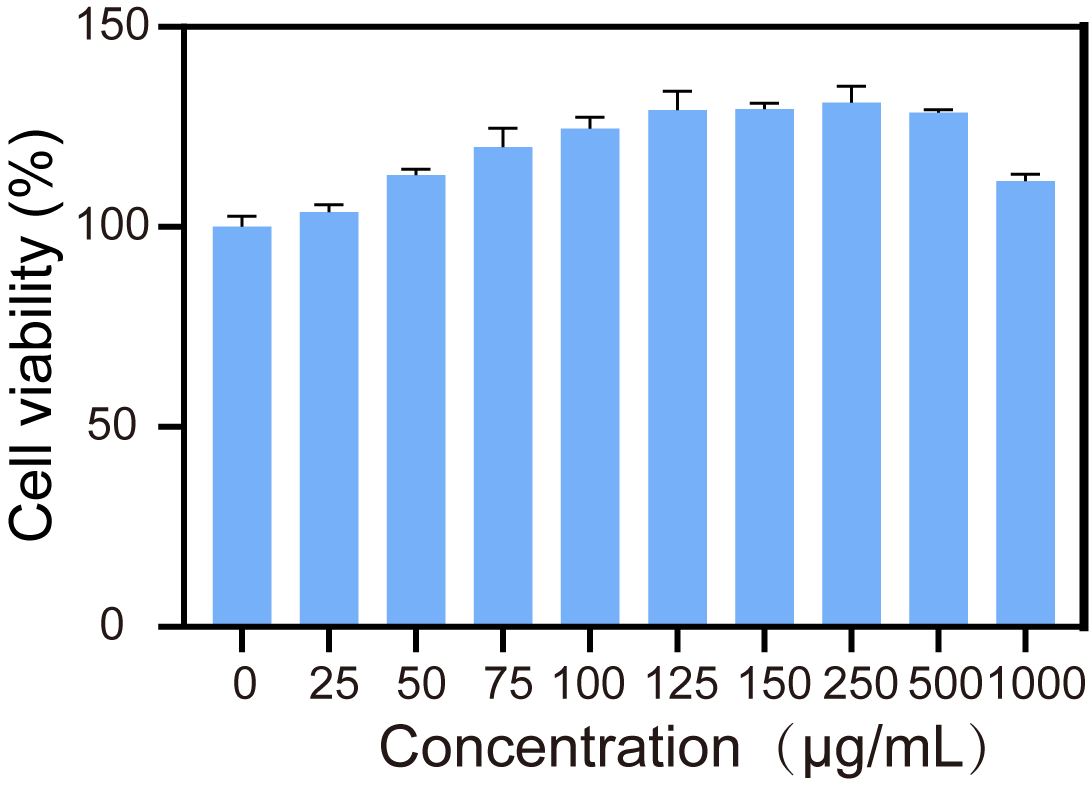


**FIGURE S10.** Relative cell viabilities of RAW 264.7 macrophages after treating with different concentrations of SMRs.


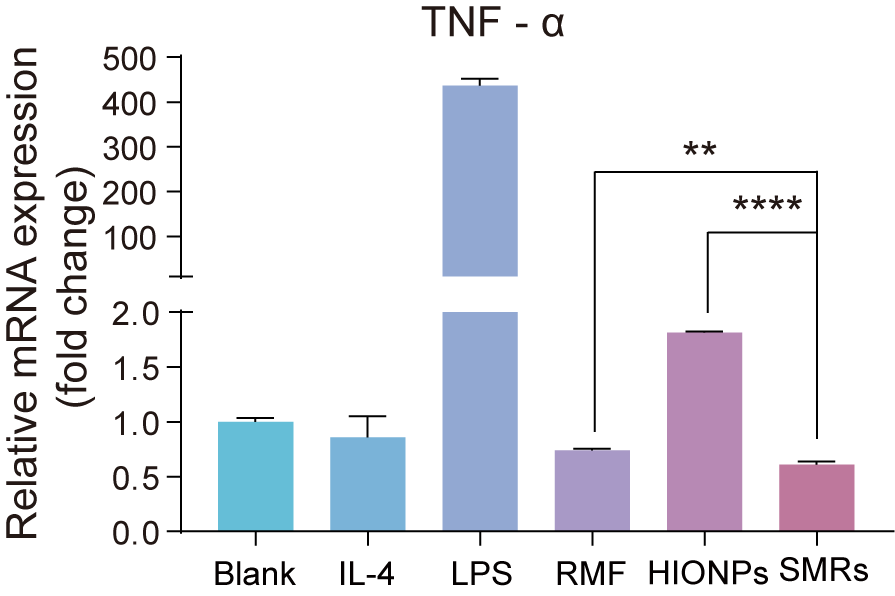


**FIGURE S11.** Gene expression level of TNF-α after treating with SMRs. Statistical significance was determined using unpaired t-test. ** p < 0.01, **** p < 0.0001. vs. control (RMF or HIONPs group).

**Supplementary Tables**

**TABLE S1.** Primer sequences for qPCR analysis.

| **Primer** | **Forward (5’-3’)** | **Reverse (5’-3’)** |
| --- | --- | --- |
| 18S | CGATCCGAGGGCCTCACTA | AGTCCCTGCCCTTTGTACACA |
| CD86 | TTGTGTGTGTTCTGGAAACGGAG | AACTTAGAGGCTGTGTTGCTGGG |
| iNOS | GGAGTGACGGCAAACATGACT | TCGATGCACAACTGGGTGAAC |
| CD206 | GCTGGCGAGCATCAAGAGTA | AGGAAACGGGAGAACCATCAC |
| PIEZO1 | TCATCATCCTTAACCACATGGTG | TGAAGACGATAGCTGTCATCCA |
| AP-1 | TTCCTCCAGTCCGAGAGCG | TGAGAAGGTCCGAGTTCTTGG |
| CCL2 | CCACAACCACCTCAAGCACT | TAAGGCATCACAGTCCGAGTC |
| TNF-α | CCACCACGCTCTTCTGTCTAC | AGGGTCTGGGCCATAGAACT |

**Supplementary movies**

Movie S1. Self-assembly of SMRs. (AVI)

Movie S2. Locomotion of SMRs under different rotation frequency in PBS. (AVI)

Movie S3. SMR followed a specified hexagon trajectory. (AVI)

Movie S4. Rotation of SMRs under magnetic field with 2Hz. (AVI)

Movie S5. SMR steered to the target cell and performed fixed-point rotation under 2Hz rotary magnetic field. (AVI)

REFERENCES

[1] Y. Wang, Y. Liu, Y. Li, D. Xu, X. Pan, Y. Chen, D. Zhou, B. Wang, H. Feng, X. Ma, *Research* **2020**, 2020, 7962024.
